# An integrative analysis reveals the prognostic value and potential functions of MTMR2 in hepatocellular carcinoma

**DOI:** 10.1038/s41598-023-46089-w

**Published:** 2023-10-31

**Authors:** Yuanqian Yao, Jiawen Lai, Yuwen Yang, Guangyao Wang, Jianlin Lv

**Affiliations:** 1 Guangxi University of Chinese Medicine, Nanning, 530000 China; 2 The First Affiliated Hospital of Guangxi University of Chinese Medicine, Nanning, 530000 China

**Keywords:** Biomarkers, Oncology, Cancer

## Abstract

Abnormal expression of myotubularin-related protein 2 (MTMR2) has been identified in certain types of cancer, leading to varying effects on tumor genesis and progression. However, the various biological significances of MTMR2 in hepatocellular carcinoma (HCC) have not been systematically and comprehensively studied. The aim of this study was to explore the role of MTMR2 in HCC. We obtained the raw data from Cancer Genome Atlas (TCGA) and Gene Expression Omnibus (GEO) databases. Afterward, we analyzed the data using R and cBioPortal. We investigated the connection between MTMR2 and its expression, prognosis, clinical significance, methylation, genetic alterations, tumor microenvironment (TME), tumor mutation burden (TMB), and drug reactivity in HCC patients. MTMR2 expression levels in HCC cells were validated through western blotting and RT-qPCR. MTMR2 exhibits high levels of expression across a wide range of cancer types, including HCC. MTMR2 is diagnostically valuable in detecting HCC, with its up-regulated expression often being indicative of poor prognosis among HCC patients. The in vitro experiments confirmed elevated MTMR2 expression in HepG2, HUH-7, and MHCC-97H cells. Univariate and multivariate Cox analysis demonstrated that MTMR2 was an independent prognostic factor in HCC patients. The cg20195272 site has the highest degree of methylation in MTMR2, and it is positively correlated with MTMR2 expression. Patients with high levels of methylation at the cg20195272 site show poor prognosis. Analysis of the TME indicates that high expression of MTMR2 is associated with elevated ESTIMATE score and that MTMR2 expression correlates positively with infiltration by resting memory CD4 T cells, activated dendritic cells, as well as several immune checkpoints. There is a negative correlation between MTMR2 expression and TMB, and drug sensitivity analyses have shown that higher MTMR2 expression is associated with lower IC50 values. This study indicates that increased expression of MTMR2 may play a crucial role in the occurrence, progression, diagnosis, prognostic prediction and drug therapy of HCC.

## Introduction

Hepatocellular carcinoma (HCC), the most common type of liver cancer, is a malignancy with significant heterogeneity and strong aggressiveness^1^. It results from an interplay between multiple factors, such as viruses and ingestion factors^2,3^, and commonly presents insidiously at onset^4^. In this context, the 5-year survival rate of HCC patients worldwide shows a falling trend^5^ in spite of the advances in treatment^4^. Patients suffering from HCC generally have a poor prognosis, which is a critical difficulty in current clinical treatment. To improve the prognosis of patients of HCC, it is necessary to look for effective and reliable signature genes that may contribute to clinical treatment.

Myotubularin-related protein 2 (MTMR2) is a member of the muscle tubulin protein family, involved in regulating many cellular biological processes such as endocytosis, membrane transport, cell division, proliferation and differentiation^6,7^. Recent studies have shown that abnormal expression of MTMR2 in certain tumors, including gastric cancer^8^ and NK/T cell lymphoma^9^, considerably promotes the development of gastric cancer by deactivating IFNγ/STAT1 signal transduction and the progression of NK/T cell lymphoma by targeting JAK1. Brenner DR et al. identified mutations in 5061 cases of squamous cell carcinoma and 33,456 control tissues using a Bayesian framework for variant prioritization and found that MTMR2 is associated with an increased risk of squamous cell carcinoma^10^. These studies indicate that MTMR2 may have a significant impact on the occurrence and development of tumors; however, the mechanism of action of MTMR2 in HCC is not clear. Therefore, the purpose of this study is to demonstrate the potential of MTMR2 as a prognostic biomarker for HCC and whether it can provide a possible choice for targeted gene therapy of tumors.

## Results

### Analysis of MTMR2 in pan-cancer

**Figure 1:**
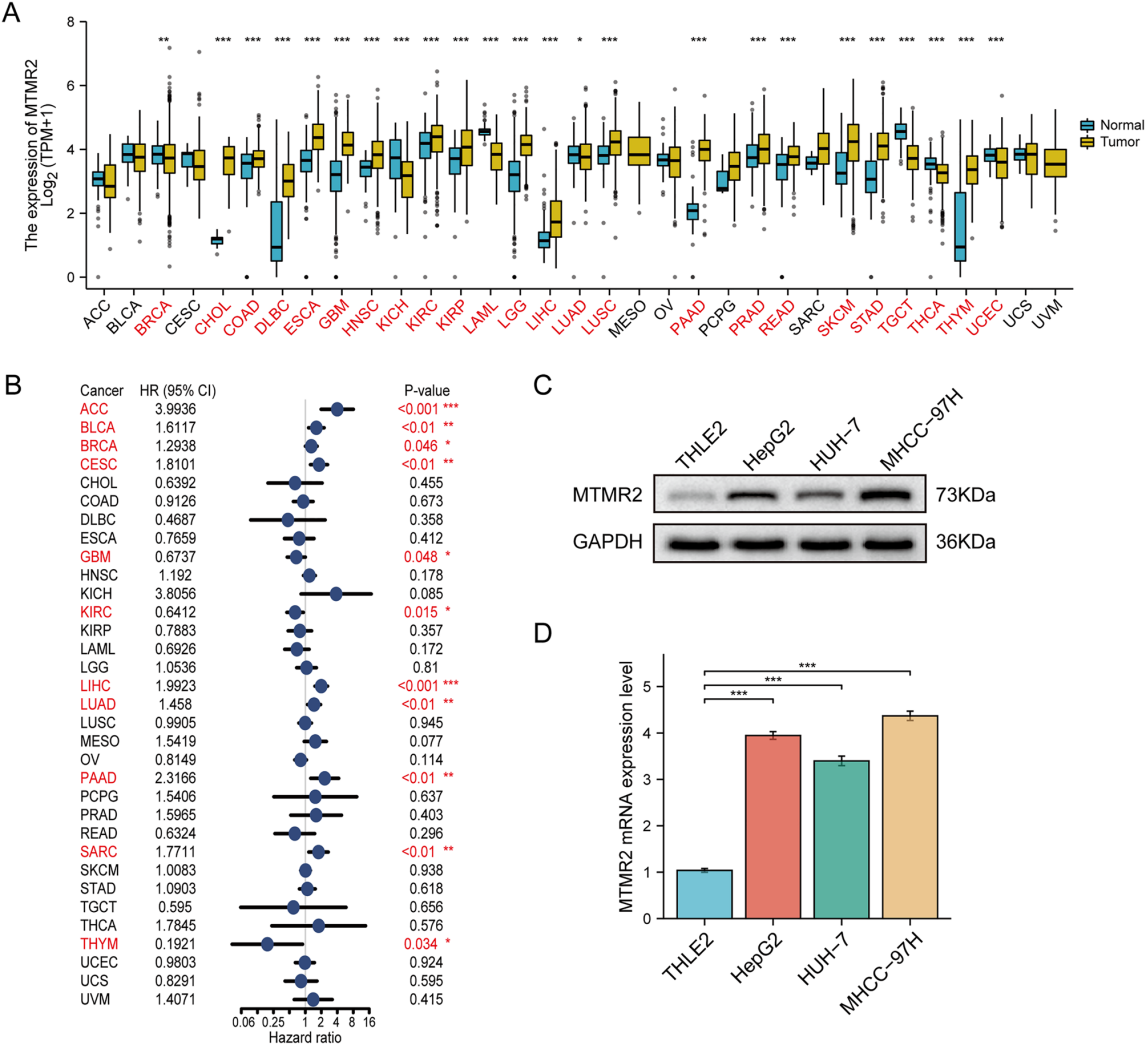
Analysis of MTMR2 in pan-cancer. (**A**) Differential expression of MTMR2 mRNA between TCGA cancer and GTEX normal tissues. (**B**) Univariate Cox regression analysis of MTMR2 prognosis in pan-cancer. (**C**) The expression of MTMR2 was verified by WB. (**D**) The expression of MTMR2 was verified by RT-qPCR (*p < 0.05, **p < 0.01, ***p < 0.001).

We analyzed the mRNA expression of MTMR2 in pan-cancer using TCGA and GTEx datasets. Out of 33 types of cancer, the expression of MTMR2 differed significantly in 24 types. The expression level of MTMR2 was significantly upregulated in cholangiocarcinoma (CHOL), colon adenocarcinoma (COAD), lymphoid neoplasm diffuse large B-cell lymphoma (DLBC), esophageal carcinoma (ESCA), glioblastoma multiforme (GBM), head and neck squamous cell carcinoma (HNSC), kidney renal clear cell carcinoma (KIRC), kidney renal papillary cell carcinoma (KIRP), brain lower grade glioma (LGG), liver hepatocellular carcinoma (LIHC), lung squamous cell carcinoma (LUSC), pancreatic adenocarcinoma (PAAD), prostate adenocarcinoma (PRAD), READ (rectum adenocarcinoma), skin cutaneous melanoma (SKCM), stomach adenocarcinoma (STAD), and thymoma (THYM) (Fig. 1A). On the other hand, the expression of MTMR2 was decreased in breast invasive carcinoma (BRCA), kidney chromophobe (KICH), acute myeloid leukemia (LAML), lung adenocarcinoma (LUAD), testicular germ cell tumors (TGCT), thyroid carcinoma (THCA), and uterine corpus endometrial carcinoma (UCEC) (Fig. 1A). It can be observed that MTMR2 exhibits widespread expression dysregulation in various cancers. Subsequently, we further assessed the relationship between MTMR2 and the survival of 33 cancer types through univariate Cox regression analysis. The results indicate that the expression level of MTMR2 is a prognostic factor for ACC, BLCA, BRCA, CESC, GBM, KIRC, LIHC, LUAD, PAAD, SARC, and THYM (*p* < 0.05) (Fig. 1B).

To validate our findings, we conducted in vitro experimental validation of MTMR2 expression using WB and RT-qPCR. The results demonstrated that the high-risk gene MTMR2 exhibited low expression in the human normal liver cell line THLE2, while it showed high expression in human HCC cell lines HepG2, HUH-7, and MHCC-97H. In summary, these experimental results confirm the reliability of our study (Fig. 1C, D).

### Genetic alteration analysis

**Figure 2:**
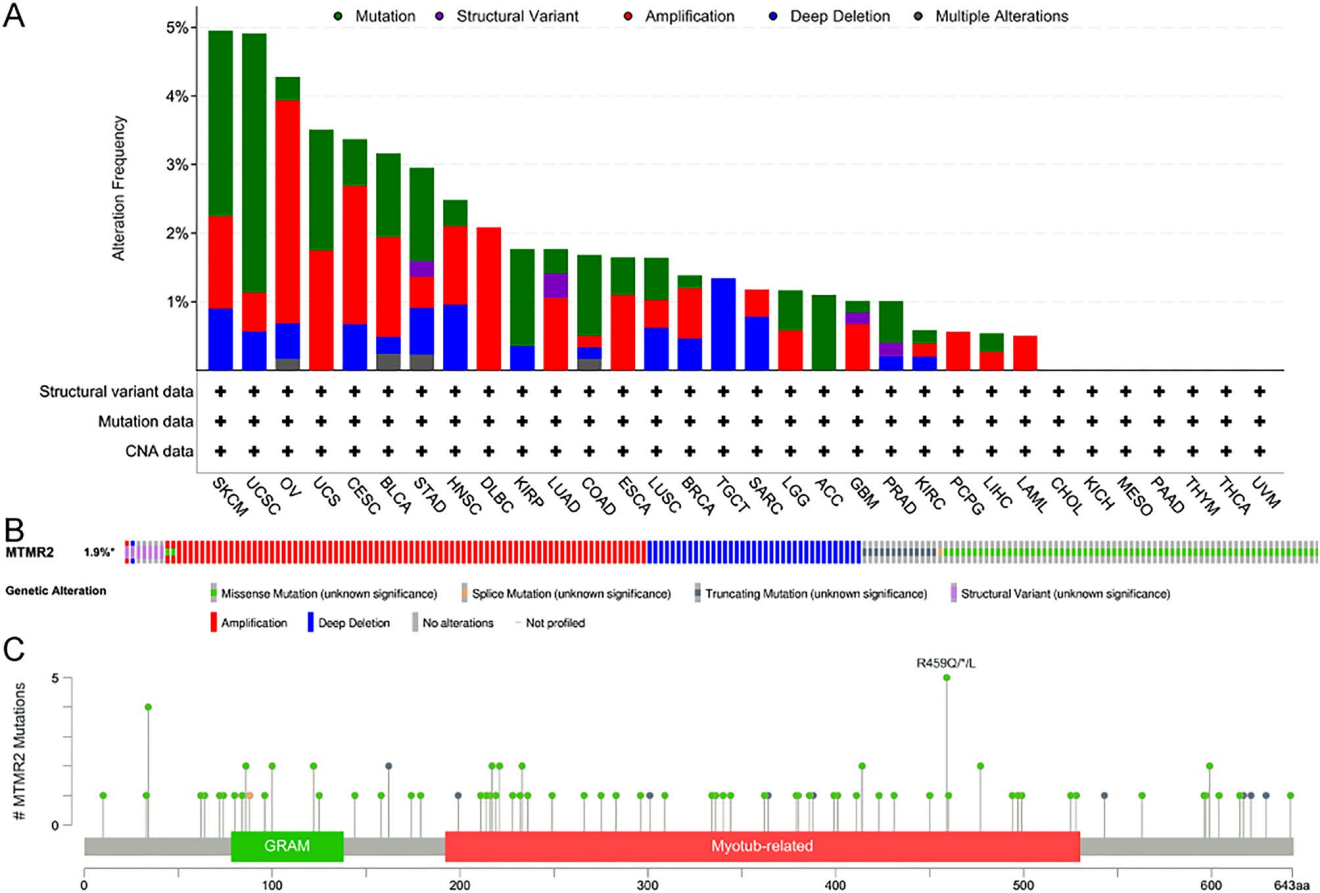
Genetic analysis of MTMR2 using cBioPortal database. (**A**) Frequency of genetic alterations in MTMR2 in the TCGA pan-cancer dataset. (**B**) Genetic alteration frequency of MTMR2. (**C**) Mutation sites of MTMR2.

We performed genetic analysis of MTMR2 on 10,967 samples from 32 studies using cBioPortal. Our results revealed that SKCM and UCSC had the highest frequency of genetic alterations, accounting for approximately 5% (Fig. 2A). The main types of genetic alterations in MTMR2 were mutation, amplification and deep deletion (Fig. 2B). A total of 98 mutation sites were identified in TCGA tumor samples, which included 77 missense mutations, 13 truncating mutations, 7 structural variants and 1 splice mutation. Among them, R459 mutation was detected in 3 cases of UCEC, 1 case of LUSC and 1 case of COAD (Fig. 2C).

### Prognostic value of MTMR2 in HCC

**Figure 3:**
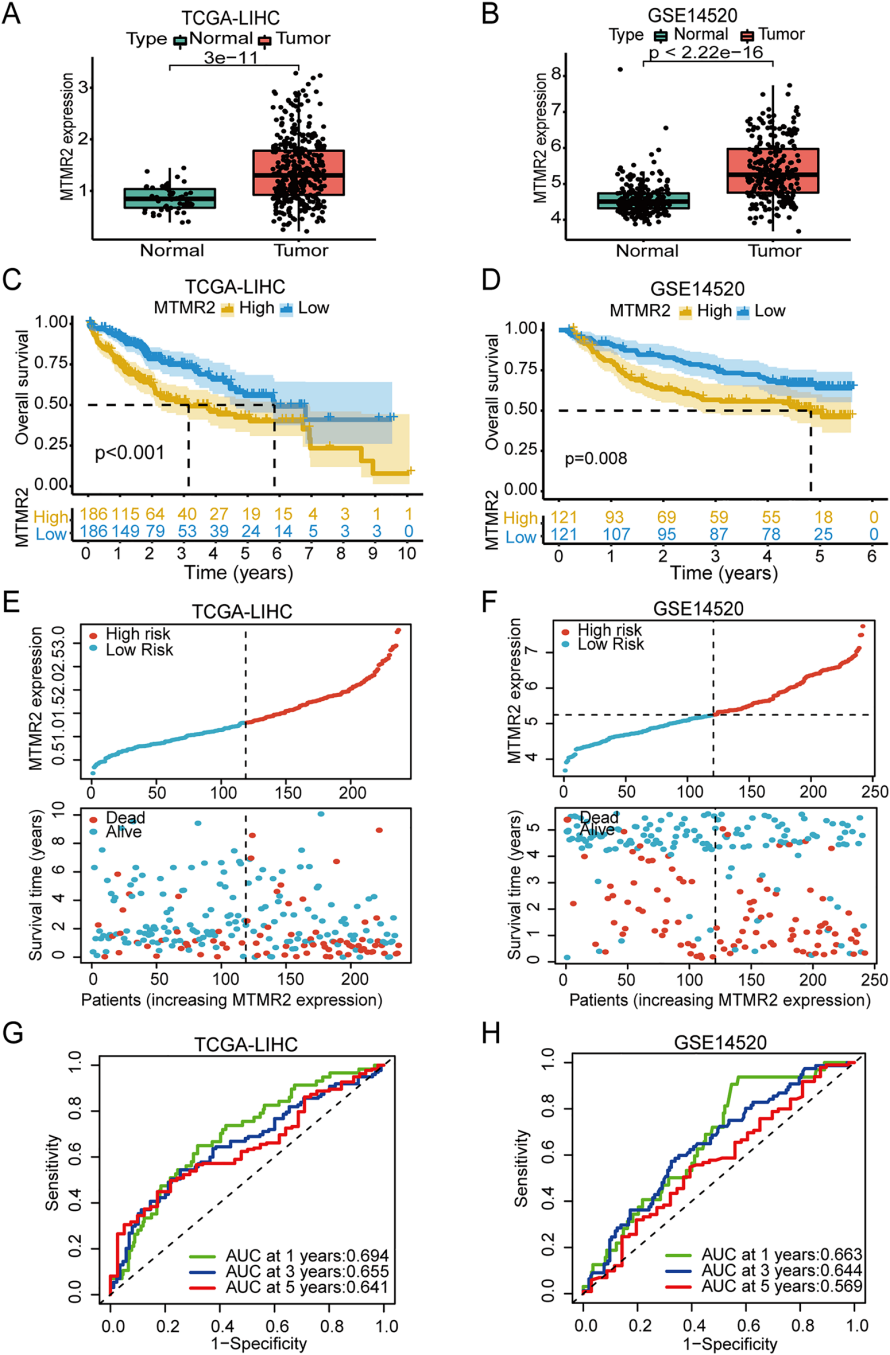
Prognostic evaluation of MTMR2 in the TCGA-LIHC and GSE14520 cohorts. (**A,B**) Expression levels of MTMR2 in normal tissues and HCC. (**C,D**) Kaplan–Meier survival curves for high and low MTMR2 expressing patients. (**E,F**) Scatter plots of the correlation between MTMR2 expression levels and survival status, survival time. (**G,H**) ROC curves for the prediction of survival period by MTMR2.

The TCGA-LIHC and GSE14520 datasets were used to evaluate the prognostic value of MTMR2 in HCC. Firstly, we evaluated the expression level of MTMR2, which was found to be higher in HCC (*p* < 0.001) (Fig. 3A, B). K–M survival curves indicated that HCC patients with low MTMR2 expression had longer OS and better prognosis compared to those with high expression of MTMR2 (*p* < 0.01) (Fig. 3C, D). Scatter plots displayed the correlation between MTMR2 expression level and survival status or time (Fig. 3E, F). To assess the predictive ability of MTMR2, we plotted ROC curves, which showed that the area under the curve (AUC) was 0.694, 0.655, and 0.641 at 1, 3, and 5 years in the TCGA-LIHC cohort, and 0.663, 0.644, and 0.569 at 1, 3, and 5 years in the GSE14520 cohort (Fig. 3G, H).

### Independent prognostic and clinical correlation analysis

**Figure 4:**
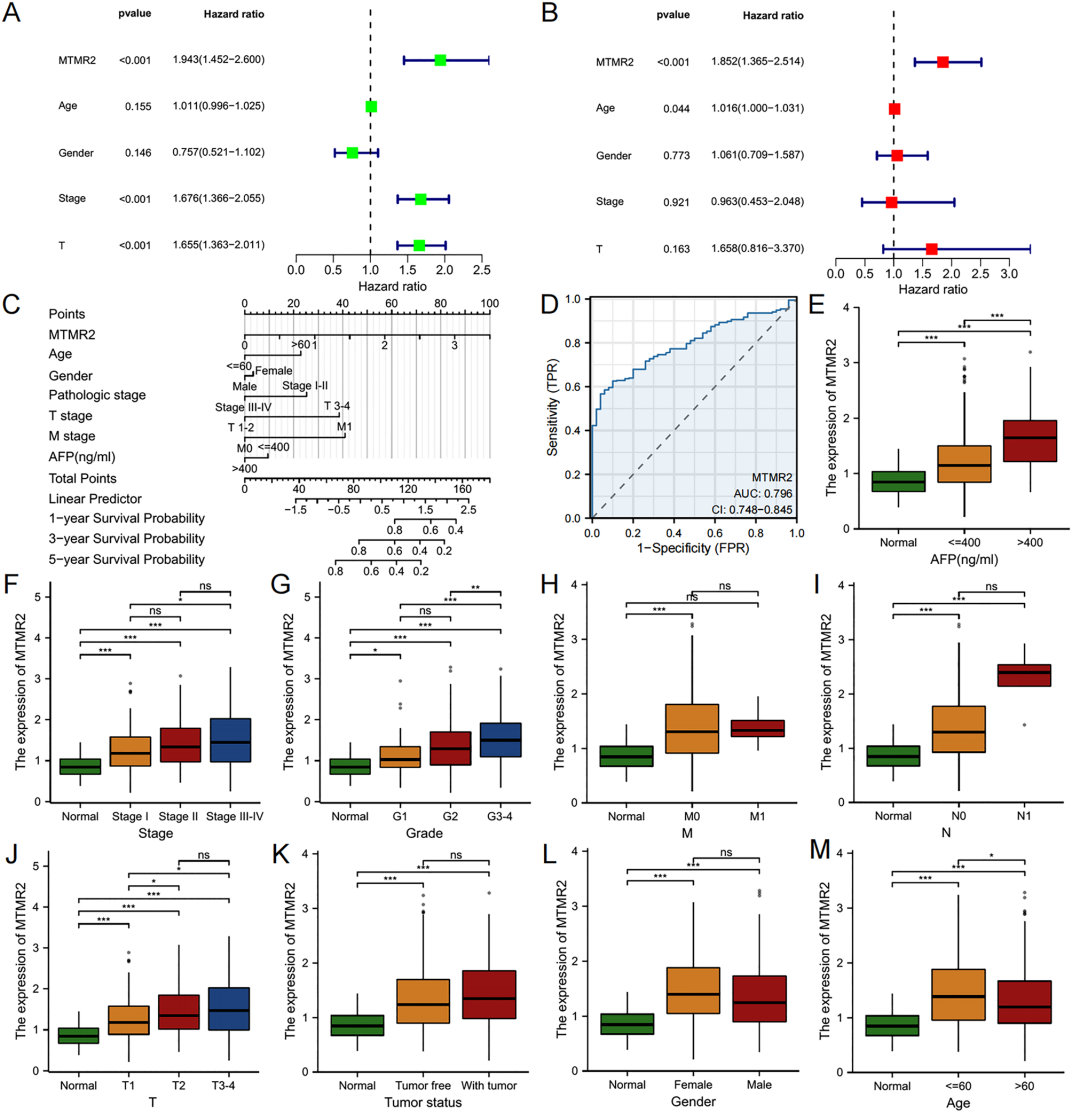
Independent prognostic and clinical correlation analysis of MTMR2 in the TCGA-LIHC cohort. (**A**) Univariate Cox regression analysis. (**B**) Multivariate Cox regression analysis. (**C**) Nomogram for predicting 1-year, 3-year, and 5-year OS of HCC patients. (**D**) ROC curves. (**E–M**) The correlation between MTMR2 expression levels and clinical features, including AFP (**E**), tumor stage (**F**), histological grade (**G**), TMN stage (**H–J**), tumor status (**K**), gender (**L**), and age (**M**).

We examined the independent prognostic value and clinical correlation of MTMR2 using the TCGA-LIHC dataset. By univariate and multivariate Cox regression analysis, we found that MTMR2 was a reliable and independent predictive factor for the prognosis of HCC (univariate: HR: 1.943, 95% CI: 1.452–2.600, *p* < 0.001; multivariate: HR 1.8521, 95% CI: 1.365–2.514, *p* < 0.001) (Fig. 4A, B). Subsequently, we construct a nomogram containing MTMR2 and clinical parameters to predict 1-year, 3-year, and 5-year OS for the entire cohort, indicating that the prognostic prediction of MTMR2 expression level was better than that of other clinical conventional parameters (Fig. 4C). The ROC curve showed that MTMR2 had high accuracy in predicting HCC (Fig. 4D). We analyzed the correlation between MTMR2 expression levels and clinical parameters, including AFP level, tumor stage, Histologic grade, TMN stage, tumor status, gender, and age (Fig. 4E–M).

### Methylation analysis of MTMR2 in HCC

**Figure 5:**
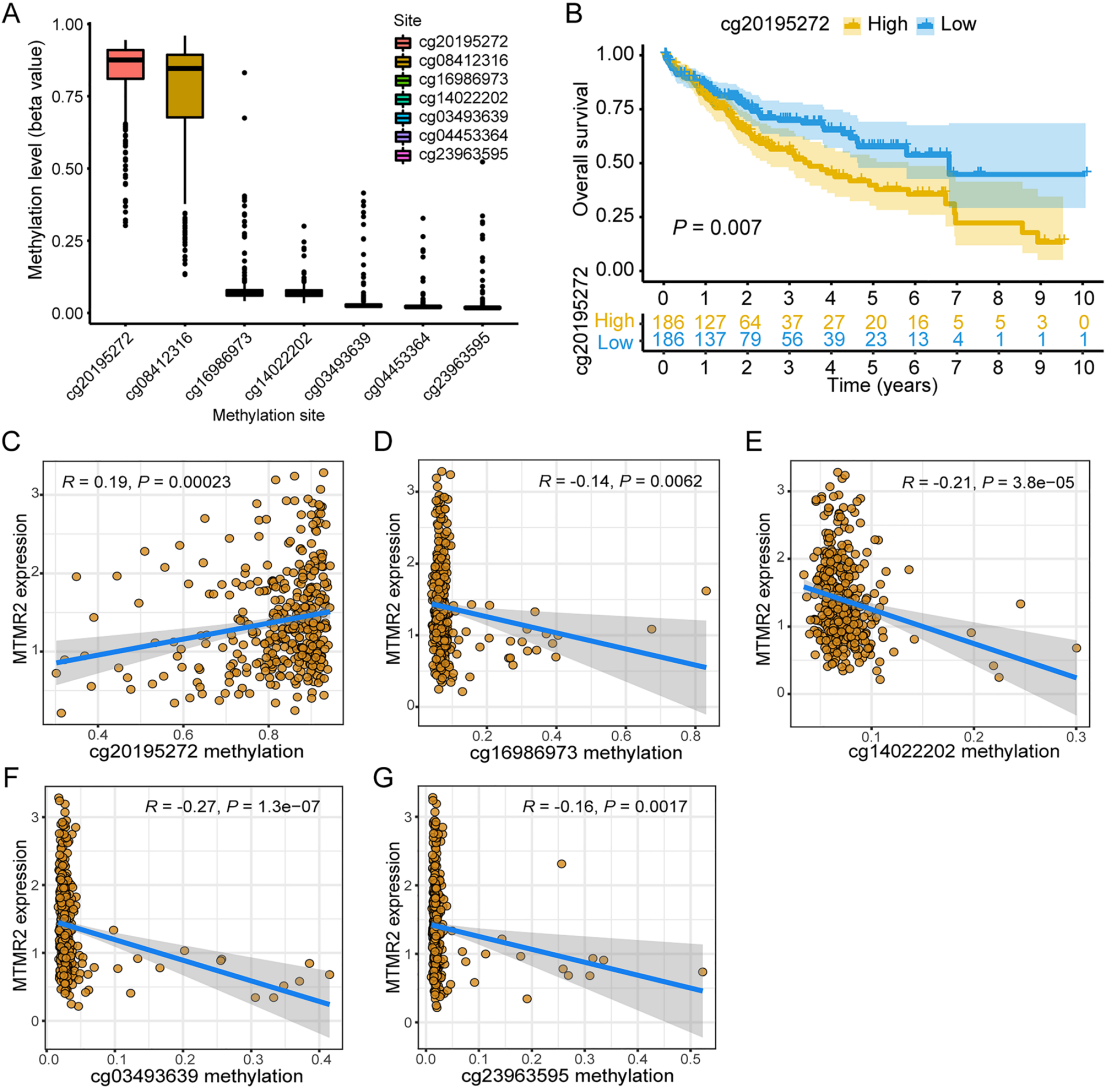
Methylation analysis of MTMR2 in HCC. (**A**) Methylation sites in the MTMR2 promoter region in HCC. (**B**) K–M survival analysis for high and low methylation groups at the cg20195272 site. (**C–G**) Methylation sites that are associated with MTMR2 expression levels.

Seven methylation sites were discovered in the MTMR2 promoter region, with cg20195272 and cg08412316 exhibiting a higher degree of methylation (Fig. 5A). Based on the median level of methylation at each site, liver cancer patients were divided into high- and low-methylation groups, and K–M analysis was subsequently used to compare the OS between the two groups. Our results demonstrated that cg20195272 was associated with the OS of HCC patients, with the low cg20195272 methylation group exhibiting a better prognosis than the high cg20195272 methylation group (*p* = 0.007) (Fig. 5B). Additionally, Spearman's correlation analysis revealed that there were five methylation sites that were correlated with MTMR2 expression levels (Fig. 5C–G).

### Drug sensitivity analysis

**Figure 6:**
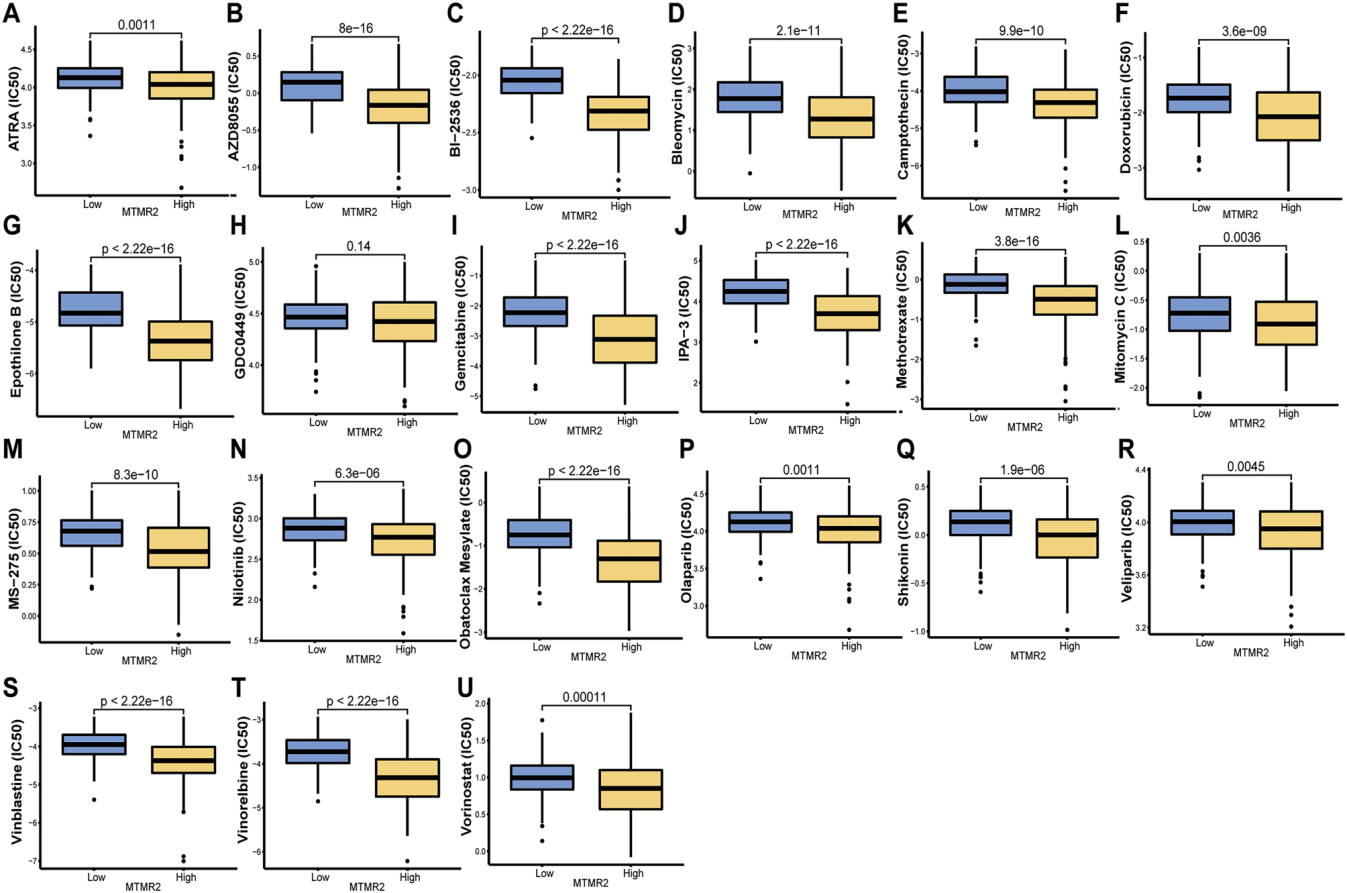
Drug sensitivity analysis. The differences in IC50 values of 21 drugs, including ATRA (**A**), AZD8055 (**B**), BI-2536 (**C**), Bleomycin (**D**), Camptothecin (**E**), Doxorubicin (**F**), Epothilone B (**G**), GDC0449 (**H**), Gemcitabine (**I**), IPA-3 (**J**), Methotrexate (**K**), Mitomycin C (**L**), MS-275 (**M**), Nilotinib (**N**), Obatoclax Mesylate (**O**), Olaparib (**P**), Shikonin (**Q**), Veliparib (**R**), Vinblastine (**S**), Vinorelbine (**T**), Vorinostat (**U**).

To investigate the association between MTMR2 expression levels and sensitivity to anticancer drugs, we compared the differences in IC50 values between the high and low expression groups of MTMR2. We selected 21 drugs with potential therapeutic effects on HCC based on the study by Wang et al.^12^. Compared with the low expression group, the high expression group had a lower predicted IC50 value, indicating that HCC patients with high MTMR2 expression were more sensitive to these drugs (Fig. 6A–U).

### Analysis of the tumor microenvironment and tumor mutation burden

**Figure 7:**
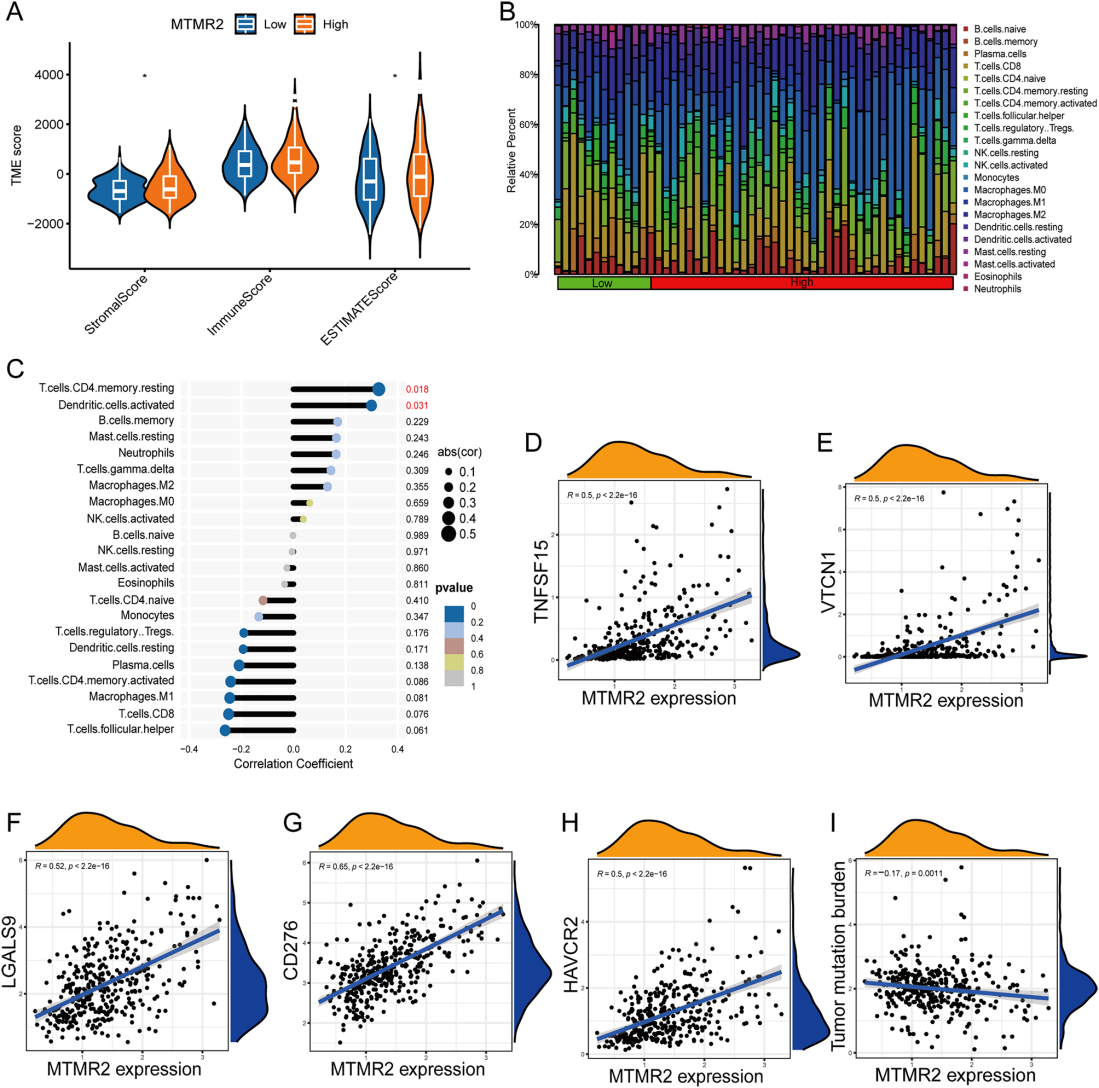
Correlation study between MTMR2 and tumor microenvironment. (**A**) Comparison of tumor microenvironment differences between high MTMR2 expression group and low MTMR2 expression group based on the ESTIMATE algorithm. (**B**) Relative content of 22 immune cells in HCC samples. (**C**) Correlation analysis between MTMR2 and 22 immune cells. (**D–H**) Correlation analysis between MTMR2 and immune checkpoint. (**I**) Correlation analysis between MTMR2 and TMB.

We used the ESTIMATE algorithm to score stromal and immune cells in HCC, and the analysis showed that the stromal score and estimate score were higher in the high MTMR2 expression group (*p* < 0.05) (Fig. 7A). Using the CIBERSORT algorithm, we calculated the relative abundance of 22 immune cells in each HCC sample (Fig. 7B). We then used Spearman's correlation analysis to examine the relationship between MTMR2 and immune cells/immune checkpoints. Our results showed that MTMR2 was positively correlated with resting memory CD4 T cells, activated dendritic cells, CD276, LGALS9, TNFSF15, VTCN1, and HAVCR2 (Fig. 7C–H). TMB is an emerging biomarker that is related to the immune response to treatment, with a higher TMB being potentially associated with a better response to immune therapy. Our correlation analysis revealed a negative correlation between MTMR2 and TMB (Fig. 7I).

### Identification of co-expressed genes and differentially expressed genes

**Figure 8:**
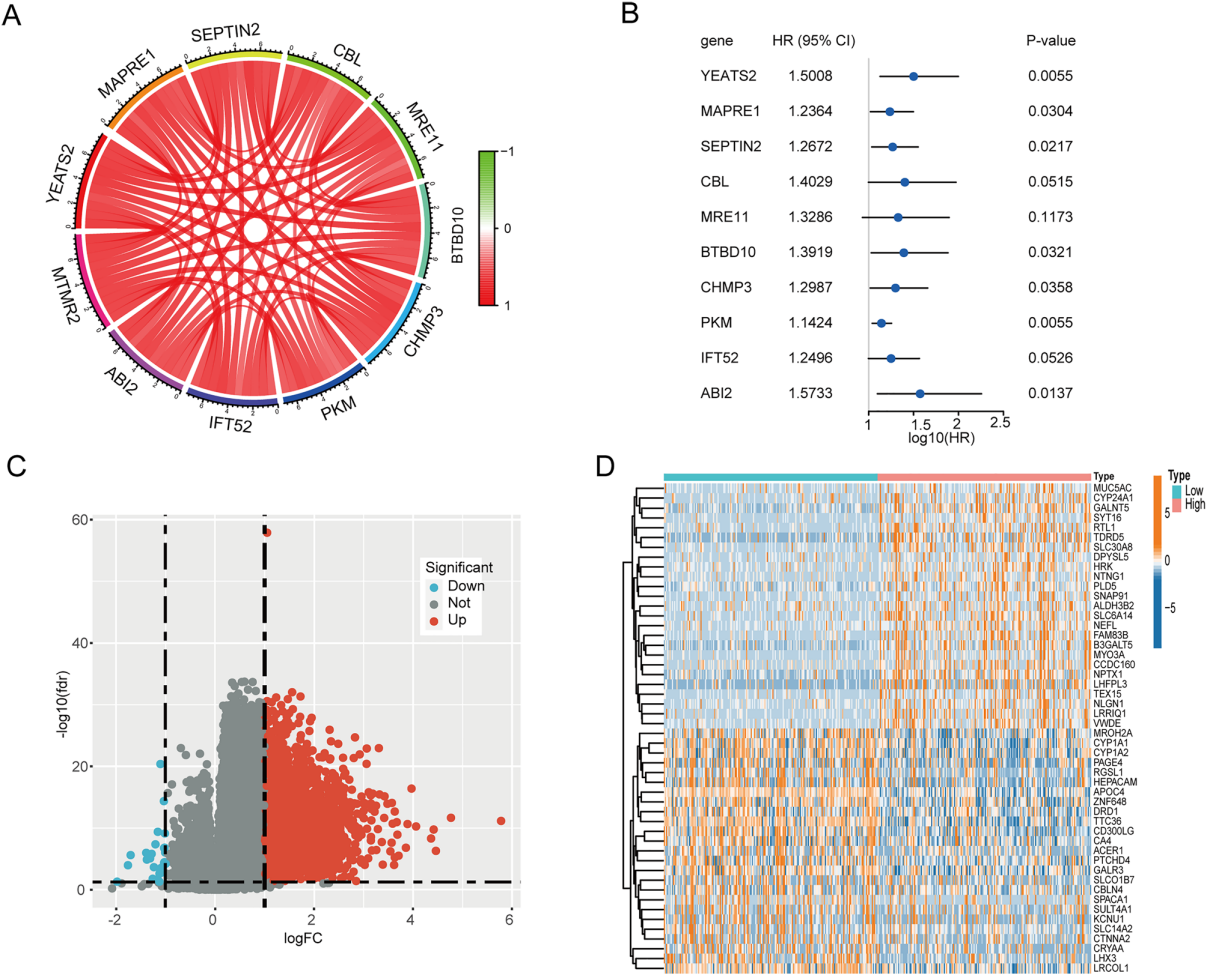
Identification of co-expressed and differentially expressed genes. (**A**) Top 10 genes co-expressed with MTMR2. (**B**) Prognostic forest plot of 10 co-expressed genes. (**C**) Volcano plot of DEGs between high and low MTMR2 expression groups. (**D**) Heatmap of expression profiles of the top 50 DEGs.

We identified 94 significantly co-expressed genes with MTMR2 through Spearman analysis (|cor|> 0.7, *p* < 0.001), and the image shows the top 10 genes with strongest correlation, all of which are positively correlated (Fig. 8A), 7 of which are associated with poor prognosis in HCC (Fig. 8B). We then performed differential expression analysis on the high MTMR2 expression group and the low MTMR2 expression group, and identified 2062 DEGs (|log2FC|> 1 and FDR < 0.05), including 2036 upregulated genes and 26 downregulated genes in HCC (Fig. 8C). The heatmap shows the expression profiles of the top 50 DEGs (Fig. 8D).

### Gene enrichment analysis

To gain a deeper understanding of the potential biological functions of DEGs and co-expressed genes in HCC, we conducted enrichment analysis for 2062 DEGs and 94 co-expressed genes separately.

The top 30 enriched GO terms for differentially expressed genes were identified as the main functions (Fig. S1A), including biological processes such as cell–cell adhesion via plasma-membrane adhesion molecules and synapse assembly, cellular components like synaptic membrane and ion channel complex, and molecular functions such as monoatomic ion channel activity and passive transmembrane transporter activity. Additionally, a total of 63 enriched KEGG pathways were identified (Fig. S1B), including prominent pathways such as Protein digestion and absorption, Calcium signaling pathway, and cAMP signaling pathway.

The top 30 enriched GO terms for co-expressed genes were identified as the main functions (Fig. S1C), including biological processes such as regulation of RNA export from nucleus and lamellipodium assembly, cellular components like actin cytoskeleton and focal adhesion, and molecular functions such as cadherin binding and SH3 domain binding. Additionally, a total of 10 enriched KEGG pathways were identified (Fig. S1D), including prominent pathways such as Regulation of actin cytoskeleton, Notch signaling pathway, and Viral carcinogenesis.

## Discussion

The liver is a vital organ involved in diverse physiological processes, such as the metabolism of macromolecules, control of endocrine growth signals, stabilization of lipid and cholesterol levels, and regulation of other physiological reactions^13^. Thus, liver-related disorders should not be taken lightly. Hepatocellular carcinoma (HCC) is a frequently occurring malignant tumor globally. Late-stage diagnoses in most patients frequently lead to poor prognoses and a reduced quality of life^14^. Consequently, researchers must continue to explore potential targets for the diagnosis and treatment of HCC. MTMR2 participates in certain signaling pathways in the human body, such as in the AKT pathway. Overexpression of MTMR2 inhibits the degradation of epidermal growth factor receptor (EGFR), thus promoting sustained activation of AKT. Through these functions, it participates in many life activities, such as signal transduction, cell cycle regulation, apoptosis, and cellular stress response^15–17^. Recent research has also demonstrated the diagnostic and therapeutic potential of MTMR2 in tumors. However, the role of MTMR2 in HCC has not yet been studied. Therefore, this study reveals the role of MTMR2 in HCC through comprehensive analysis.

In this study, we analyzed the TCGA and GTEx databases and found that MTMR2 is significantly overexpressed in various cancer types, including HCC, and that increased expression of MTMR2 is associated with poor patient prognosis. We validated the differential expression and prognostic value of MTMR2 in HCC using an independent cohort, GSE14520. Additionally, analysis of the TCGA dataset showed that increased MTMR2 expression in HCC is significantly correlated with patient age, pathological stage, histological grade, T stage, and AFP concentration. Multivariate analysis revealed that MTMR2 is an independent prognostic factor for HCC patients' overall survival. Co-expression analysis revealed that MTMR2 is positively correlated with the top 10 most strongly related genes, among which 7 genes are significantly associated with poor prognosis of HCC. These genes may work synergistically to promote the progression of HCC. Thus, MTMR2 may serve as a diagnostic and prognostic molecular biomarker for HCC patients.

Aberrant DNA methylation has been recognized as a common epigenetic alteration in human cancers^18^. Therefore, DNA methylation has received increasing attention in the study of tumor occurrence, early diagnosis, and prognostic assessment. Studies have shown that DNA methylation plays an important role in the occurrence and development of tumors and is considered one of the important mechanisms of tumor development and progression^19^. Our study revealed that the expression level of MTMR2 was negatively correlated with the methylation levels of the cg16986973, cg14022202, cg03493639, and cg23963595 sites, consistent with the widely recognized role of DNA methylation in gene expression regulation, where gene methylation often suppresses gene expression^20^. Interestingly, our research indicated a positive correlation between the methylation level of the cg20195272 site and MTMR2 expression, which may be related to the complex regulatory mechanisms of DNA methylation. In fact, there are certain exceptional cases, such as the MLH1 gene, which is an essential component of the DNA mismatch repair system. Studies have found that highly methylated MLH1 promoter regions are associated with elevated expression in certain tumors^21,22^. This is because in certain situations, highly methylated MLH1 may attract methylation-specific proteins, such as MECP2 and MBD2, which can bind to methylated DNA and facilitate gene transcription^22^. Furthermore, the high methylation of the MLH1 gene promoter region may not be uniform. In certain regions, especially at the edges of CpG islands, there may be unmethylated or low-methylated regions that allow transcription factors to bind and promote gene expression^21^. A recent study has also indicated that many transcription factors preferentially bind to highly methylated sequences of certain genes, potentially promoting their high expression^23^. These findings suggest that the relationship between methylation and gene expression is influenced by multiple factors and cannot be confined to a single regulatory model. Our study suggests that the expression of MTMR2 in HCC may be subject to complex methylation regulation, warranting further investigation.

The TME is a highly complex structure composed of cellular components such as cancer-associated fibroblasts (CAFs), immune cells, endothelial cells, and adipocytes, as well as non-cellular components like the extracellular matrix (ECM)^24^. Persistent stimulation by tumor antigens leads to exhaustion and reprogramming of effector cells within the TME, promoting tumor immune evasion and ultimately driving tumor progression^25,26^. In recent years, there has been rapid development in the research and application of immunotherapy, yielding substantial achievements in many malignant tumors. The crucial role of TME in tumor progression and therapy has also garnered increasing attention^27,28^. In this study, we found that patients with high MTMR2 expression had higher stromal scores and tumor purity scores. Tumor stromal cell infiltration is an important factor promoting the progression of HCC^29,30^. Further immune infiltration analysis showed that MTMR2 is significantly correlated with the infiltration levels of CD4+ memory resting T cells and activated dendritic cells, and is positively correlated with immune checkpoint markers CD276, LGALS9, TNFSF15, VTCN1, and HAVCR2 (*p* < 0.05). In addition, we also found that MTMR2 is negatively correlated with TMB. TMB refers to the total number of non-synonymous mutations in the coding region of the tumor genome and is a new emerging biomarker that reflects the effectiveness of immune checkpoint inhibitors (ICIs)^31^. Theoretically, a higher number of mutations can lead to an increase in the recognition of neoantigens and immunogens, promoting T-cell responses against tumor cells. Therefore, a higher TMB represents greater intratumoral heterogeneity, which may result in an increased response to immunotherapy^32^. We speculate that MTMR2 may promote immune escape and reduce the effectiveness of immunotherapy by increasing co-expression with immune checkpoints and inhibiting tumor cell mutation rates. Therefore, MTMR2 may be a potential target for immunotherapy in HCC.

Systemic drug therapy is an important treatment modality for HCC^33^. HCC typically presents as an "immunologically cold" state, protecting cancer cells from the attacks of infiltrating lymphocytes, leading to poor immune therapeutic response^34^. Therefore, it is necessary to identify the mechanism of drug resistance. This study explored the potential correlation between MTMR2 expression and the IC50 value of anti-cancer compounds. We found that in 21 drugs with therapeutic potential for HCC, the IC50 values of the high MTMR2 expression group were lower, indicating that HCC patients with high MTMR2 expression were more sensitive to these 21 drugs. Nevertheless, more clinical evidence is still needed to evaluate the impact of these drugs on tumor treatment.

The study offers novel insights and materials to individualize clinical management plans for liver cancer patients. However, the study's limitations should be acknowledged. Firstly, the research only conducted in vitro cell experiments, and it lacked validation of clinical specimens. Additionally, the study employed a retrospective design rather than a prospective one. However, this study was conducted in two separate independent cohorts, thus the outcomes remain dependable and satisfactory. Subsequently, forthcoming research implementing prospective clinical trials and exploring mechanisms are necessary to further verify the present discoveries.

## Conclusion

In summary, our study suggests that MTMR2 expression is upregulated in HCC, which is associated with unfavorable prognosis. Furthermore, it demonstrates the correlation between MTMR2 expression and clinical factors, methylation, TME, TMB, and drug sensitivity of HCC, and explores co-expression genes. MTMR2 can serve as a prognostic biomarker and a potential therapeutic target for HCC.

## Materials and methods

### Data collection and gene expression analysis

Obtain mRNA expression data of both normal and cancerous samples of 33 cancer types from the TCGA database (https://tcga-data.nci.nih.gov/tcga/) and the GTEx database (https://www.gtexportal.org/). Moreover, obtain methylation data and tumor mutation data specifically for TCGA-LIHC (including 374 HCC tissue samples and 50 normal liver tissue samples). Additionally, we downloaded the GSE14520 dataset (including 247 HCC tissue samples and 241 normal liver tissue samples) from the GEO database (https://www.ncbi.nlm.nih.gov/geo/)^11^. Wilcoxon rank-sum test was used to analyze the differential expression of MTMR2 in various cancer types, as well as tumor and normal tissues within GSE14520. The R software was used for analysis.

### Survival prognosis analysis and clinical correlation analysis

Using univariate Cox regression analysis to assess the survival impact of MTMR2 across 33 types of cancers, displayed through a forest plot. Additionally, we further analyzed the TCGA-LIHC and GSE14520 datasets using the R packages "survival" and "survminer" to draw Kaplan–Meier (KM) plots and compare the OS of high and low MTMR2 expression levels in HCC patients. We used the R package "timeROC" to draw receiver operating characteristic (ROC) curves and evaluate the sensitivity and accuracy of MTMR2 in predicting HCC patients. We used univariable and multivariable Cox regression analysis to evaluate whether MTMR2 is an independent factor in predicting the OS of HCC patients. We performed Kruskal–Wallis tests to analyze the correlation of MTMR2 with different clinical features and subtypes.

### Genetic alteration analysis

We analyzed the genetic alterations of MTMR2 using cBioPortal (https://www.cbioportal.org/). Based on the "TCGA Pan Cancer Atlas Studies" dataset, we calculated the frequency and copy number changes of MTMR2 gene mutations in the "Cancer Types Summary" module. We used the "Mutations" module to construct a mutation site map for MTMR2.

### Methylation analysis

We used Spearman correlation analysis to determine the relationship between the MTMR2 promoter methylation sites and its mRNA expression. We divided HCC patients into high and low methylation groups according to the median level of MTMR2 methylation and evaluated the prognostic value of MTMR2 promoter methylation sites through K–M survival analysis.

### Drug sensitivity, tumor microenvironment (TME), and tumor mutation burden (TMB) analysis

We used the R package "pRRophetic" to predict the sensitivity of HCC patients to anticancer drugs, and used the Wilcoxon rank sum test to compare the half maximal inhibitory concentration (IC50) of drugs between high and low MTMR2 expression groups. We used the R package "ESTIMATE" to evaluate the stromal score, immune score, and estimate score of HCC patients, and used the Wilcoxon rank sum test to compare the differences in TME between high and low MTMR2 expression groups. We used the CIBERSORT algorithm to evaluate the infiltration ratio of 22 immune cells in each HCC patient. We used Spearman correlation analysis to evaluate the correlation of MTMR2 with TMB, immune cells, and immune checkpoints.

### Screening co-expressed genes and differentially expressed genes

Using the TCGA-LIHC dataset, we identified the co-expressed genes of MTMR2 through Spearman correlation analysis (|cor|> 0.7, *p* < 0.001). We used the R package "limma" to identify differentially expressed genes (DEGs) between the high and low MTMR2 expression groups, using |log2FC|> 1 and FDR < 0.05 as filtering conditions. Gene ontology (GO) and Kyoto Encyclopedia of Genes and Genomes (KEGG) enrichment analysis of DEGs and co-expressed genes was performed using the R package "clusterProfiler".

### Cell culture

Human HCC cells (HepG2, HUH-7 and MHCC-97H) were cultured in high-glucose DMEM (Gibco, USA), while normal liver cells (THLE2) were cultured in BEGM (Thermo fisher scientific, USA). In both cases, the culture medium was supplemented with 10% fetal bovine serum, and the cells were maintained in a 5% CO2 incubator (Heal force, China) at a constant temperature of 37 ℃.

### Western blotting

Total protein was extracted from cells (HepG2, HUH-7, MHCC-97H and THLE2) lysed in RIPA buffer (Solarbio, China) and quantified using a BCA kit (Solarbio, China). The expression of MTMR2 (1:1000, A8993, ABclonal, China) in the four cell lines was detected via western blotting (WB).

### Real-time reverse transcription polymerase chain reaction

Real-time reverse transcription polymerase chain reaction (qPCR) was performed to examine the mRNA expression of MTMR2 in HepG2, HUH-7, MHCC-97H and THLE2 cells. Total RNA was extracted from cells using the Promega kit and quantified. cDNA was synthesised using a reverse transcription kit. The PCR conditions were as follows: 40 cycles of pre-denaturation (95 ℃, 2 min), denaturation (95 ℃, 30 s), annealing (60 ℃, 30 s) and extension (72 ℃, 30 s). GAPDH was used as an internal control, and the relative expression level of MTMR2 was evaluated using the 2^−△△Ct^ method. Primer sequences (Bioengineering Co., Ltd, Shanghai): MTMR2, forward 5'-TCCTTAGCCTCCTTCGACCT-3' and reverse 5'-TCCAGGTGCACAAGACAGAC-3'; GAPDH, forward 5'-TGCACCACCAACTGCTTAGC-3' and reverse 5'- GGCATGGACTGTGGTCATGAG-3'.

### Supplementary Information

Supplementary Figures.

## Data Availability

The datasets used and analyzed during the current study are available from The Cancer Genome Atlas (TCGA, https://portal.gdc.cancer.gov/), Genotype-Tissue Expression (GTEx, https://www.gtexportal.org/), and Gene expression Omnibus (GEO, https://www.ncbi.nlm.nih.gov/geo/).
