# Supplementary material for: An integrative analysis reveals the prognostic value and potential functions of MTMR2 in hepatocellular carcinoma
Source: Sci Rep. 2023 Oct 31;13:18701. doi: 10.1038/s41598-023-46089-w (PMC10618242; doi:10.1038/s41598-023-46089-w)

**Supplementary Figure**


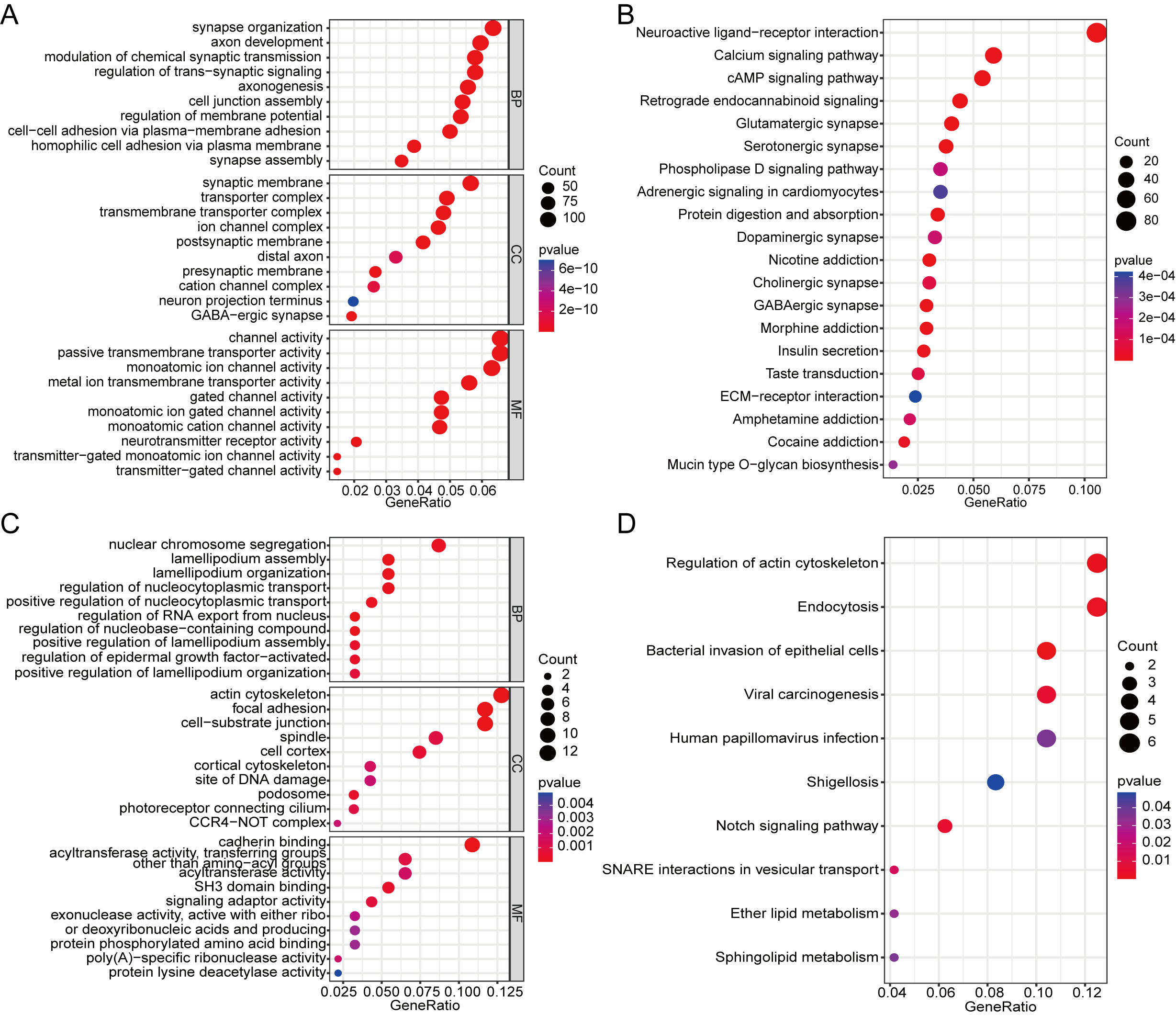


**Figure S1.** Enrichment analysis of DEGs and co-expressed genes. (A) GO enrichment analysis of DEGs. (B) KEGG enrichment analysis of DEGs. (C) GO enrichment analysis of co-expressed genes. (D) KEGG enrichment analysis of co-expressed genes.

**Unprocessed WB original images**

**MTMR2:**


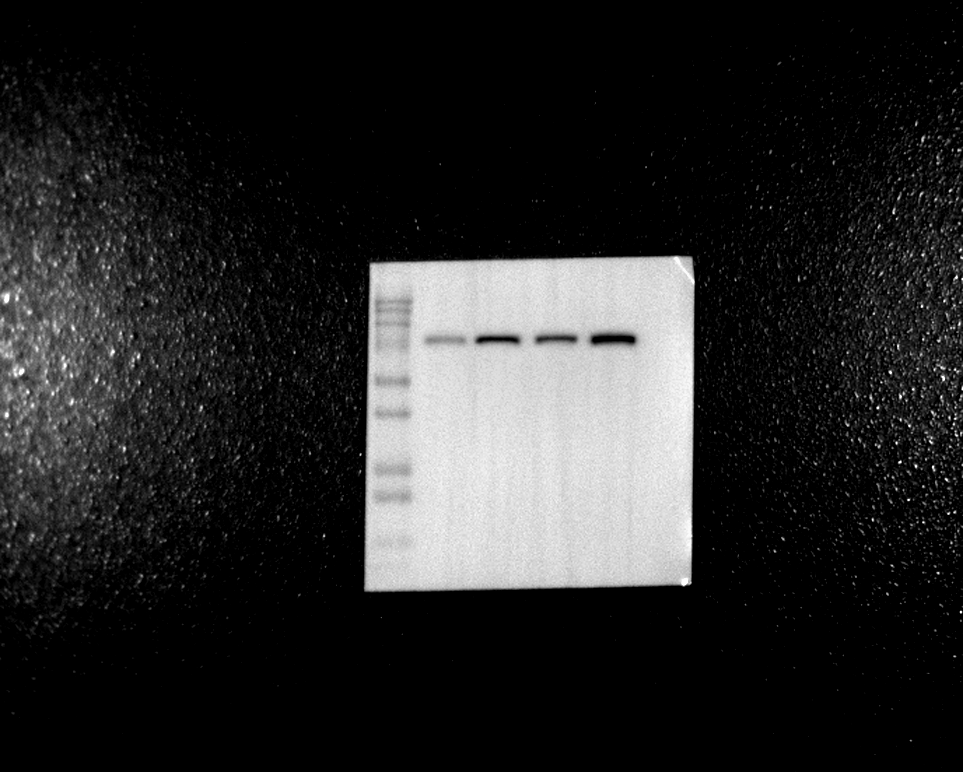


**GAPDH:**


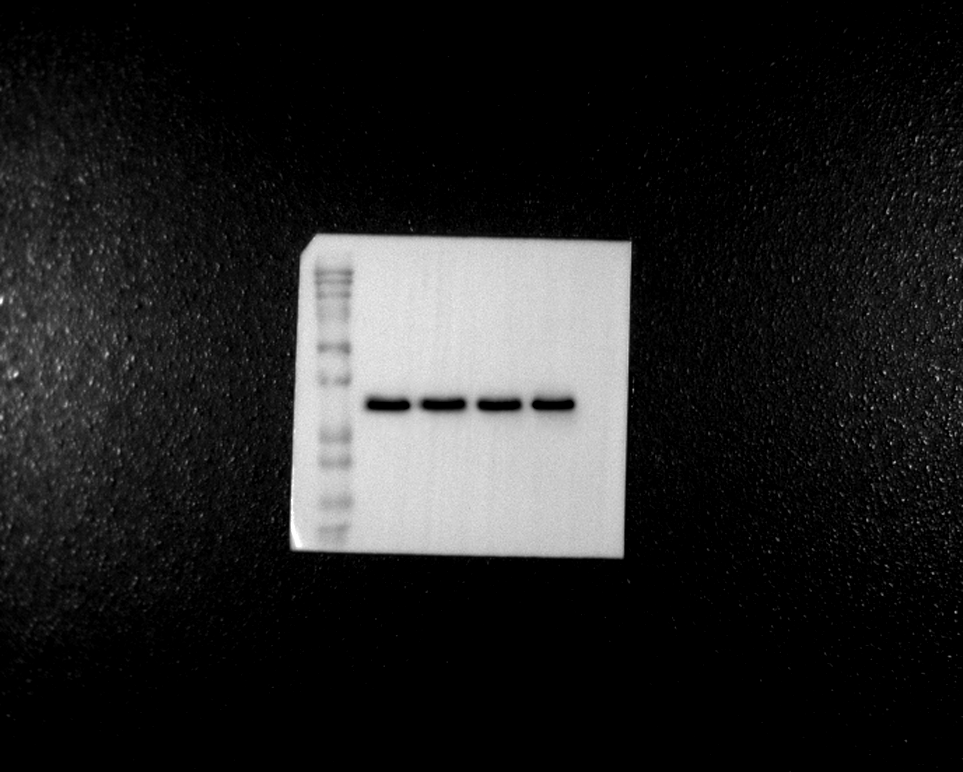

Supplement: Supplementary file 1 — Supplementary Figures. [file 41598_2023_46089_MOESM1_ESM.docx]
